# Hyperosmia, ectrodactyly, mild intellectual disability, and other defects in a male patient with an X-linked partial microduplication and overexpression of the *KAL1* gene

**DOI:** 10.1007/s13353-014-0252-7

**Published:** 2014-10-23

**Authors:** Anna Sowińska-Seidler, Monika Piwecka, Ewelina Olech, Magdalena Socha, Anna Latos-Bieleńska, Aleksander Jamsheer

**Affiliations:** 1 Department of Medical Genetics, Poznan University of Medical Sciences, Rokietnicka 8 Street, 60-806 Poznan, Poland; 2 Institute of Bioorganic Chemistry, Polish Academy of Sciences, Noskowskiego 12/14 Street, 61-704 Poznan, Poland; 3 NZOZ Center for Medical Genetics GENESIS, 4 Grudzieniec Street, 60-601 Poznan, Poland

**Keywords:** *KAL1*, Duplication, Anosmin-1, Hyperosmia, SHFM, Ectrodactyly

## Abstract

Loss-of-function mutations of the *KAL1* gene are a known cause of Kallmann syndrome, a disorder characterized by the coexistence of hypogonadotropic hypogonadism and anosmia/hiposmia. On the other hand, neither complete nor partial duplications of *KAL1* have been reported in the literature; thus, clinical symptoms associated with such alterations remain unknown. Ectrodactyly is a clinically and genetically heterogeneous abnormality presenting with hypoplasia of the central rays of the extremity, which, in around 68 % of cases, has unknown underlying molecular defect. In this paper, we report on a sporadic male patient manifesting hyperosmia and ectrodactyly accompanied by additional symptoms involving mild intellectual disability, unilateral hearing loss, genital anomalies, stocky build, and facial dysmorphism. Using a combination of high-resolution array comparative genomic hybridization (array CGH) and breakpoint analysis, we detected a hemizygous tandem duplication of 110,967 bp on Xp22.31, encompassing the promoter region and the first two exons of *KAL1*. In order to confirm pathogenicity of the duplication, we tested the level of *KAL1* transcript in blood lymphocytes, showing 79 times higher expression in the proband compared to controls. We, therefore, hypothesize that olfactory hypersensitivity in our proband directly results from *KAL1* overproduction. Additionally, a literature review allowed us to conclude that *KAL1* protein at high levels may interfere with FGFR1 signaling activity, most probably indirectly giving rise to ectrodactyly, intellectual disability, and genital anomalies. Noteworthy, those symptoms overlap with Hartsfield syndrome caused by FGFR1 loss-of-function mutations. To conclude, our paper highlights the role of *KAL1* in embryogenesis and provides data on the contribution of *KAL1* overexpression to human pathology.

## Introduction

Congenital limb anomalies categorized as split hand/foot malformation (SHFM; OMIM 183600) are phenotypically heterogeneous disorders; however, the common characteristic feature shared by all types of SHFM involves the absence or hypoplasia of the central ray(s) of the autopod. In its most severe form, the aplasia of both central and preaxial rays of the hands and/or feet leads to monodactyly. Clinical heterogeneity of SHFM (also referred to as ectrodactyly) results mostly from variable expressivity of the feature, which often manifests inconstantly in closely related patients or even in different limbs of a single individual (Basel et al. 2006). Ectrodactyly occurs either as an isolated trait or part of a multiple congenital anomaly syndrome. The malformation is caused by a failure in the development or maintenance of the median apical ectodermal ridge (AER), a group of cells located in the most distal portion of the developing limb bud (Duijf et al. 2003). To date, seven loci associated with non-syndromic SHFM have been described. These include: SHFM1 (OMIM 183600, caused by various rearrangements in the 7q21.3 locus or point mutations in *DLX5*), SHFM2 (OMIM 313350, located in Xq26), SHFM3 (OMIM 246500, resulting from tandem duplications of the 10q24-q25 locus), SHFM4 (OMIM 605289, caused by heterozygous mutations in *TP63*), SHFM5 (OMIM 606708, linked to deletions in the 2q31 locus), SHFM6 (OMIM 22530, associated with homozygous mutations in *WNT10B*), as well as the most common form, i.e., SHFM/SHFLD3; MIM 612576 (caused by 17p13.3 duplication encompassing *BHLHA9*) (Scherer et al. 1994; Faiyaz-Ul-Haque et al. 2005; Gurrieri et al. 1996; van Bokhoven et al. 2001; Klopocki et al. 2012; Goodman et al. 2002; Sowińska-Seidler et al. 2014). Despite recent progress and dissemination of molecular diagnostic and research techniques, the genetic cause of the majority of SHFM cases remains unresolved (Klopocki and Mundlos 2011; Klopocki et al. 2012; Biegański et al. 2012; Tayebi et al. 2014).

Structural copy number variations (CNVs) are a known cause of human limb malformations, and the number of studies associating various CNVs with developmental limb disorders is constantly growing (Klopocki and Mundlos 2011; Klopocki et al. 2012; Jamsheer et al. 2013). The contribution of submicroscopic genomic rearrangements to the pathogenesis of human malformations can occur via several mechanisms. The most common involves alteration of the copy number of a dosage-sensitive gene or gene cluster. In this setting, deletions or duplications simply lead to the haploinsufficiency or overexpression of a gene, respectively. In some cases, deletions and duplications of the same dosage-susceptible gene give rise to different clinical conditions, as previously shown, for example, for *SOX9*, *MECP2*, or *PMP22* (Benko et al. 2009; Kurth et al. 2009; Meins et al. 2005; Van Esch et al. 2005; Patel et al. 1992; Valentijn et al. 1995; Chance et al. 1993). Alternatively, CNVs can also cause position effects and disturb gene expression by changing the number or location of regulatory elements or by altering the entire regulatory landscape of a target gene or gene cluster (Klopocki and Mundlos 2011).

The *KAL1* gene is involved in embryonic development of the kidney and human central nervous system, including the spinal cord, olfactory bulbs, olfactory nerves, and retina (Duke et al. 1995). While *KAL1* loss-of-function alterations (i.e., deletions and point mutations) are a known cause of X-linked Kallmann syndrome (KS), which is characterized by the association of hypogonadotropic hypogonadism (HH) and anosmia/hiposmia, complete or partial duplications of this gene have not been reported in the literature. Thus, clinical symptoms associated with *KAL1* duplications and/or increased gene expression remain unknown.

In this paper, we report on a sporadic male patient presenting with SHFM and hyperosmia accompanied with additional congenital abnormalities, most likely resulting from a partial duplication of *KAL1* and significant overexpression of this gene. Our paper provides further evidence on the contribution of CNVs to congenital malformations in humans and highlights the role of *KAL1* in embryonic development.

## Methods

All patients agreed to participate in this study and informed consent was obtained from all subjects or their legal guardians prior to genetic testing. Ethics approval was granted by the Institutional Review Board of the Poznan University of Medical Sciences.

### Array comparative genomic hybridization (array CGH)

Genomic DNA was extracted from peripheral blood leukocytes according to the salting-out method. Array comparative genomic hybridization (array CGH) was carried out with the use of the 1.4M NimbleGen oligonucleotide CGH array (Roche NimbleGen), according to standard protocols provided by the manufacturer. Analysis was done with Deva software (Roche NimbleGen). The analysis settings were as follows: aberration algorithm: ADM-2; threshold: 6.0; window size: 0.2 Mb; filter: five probes, log_2_ ratio = 0.29. The genomic profile was visualized by the SignalMap Software (SignalMap, NimbleGen Systems, Inc.).

### Quantitative real-time polymerase chain reaction (qPCR)

In order to confirm array CGH results and narrow down the genomic coordinates of the rearrangement, we performed a quantitative real-time polymerase chain reaction (qPCR) using a ViiA™ 7 Real-Time thermal cycler (Applied Biosystems). The qPCR assay was designed to determine the number of copies in the vicinity of both 5′ and 3′ ends of the duplicated Xp22.31 segment. The test was carried out with a set of six and three primer pairs, respectively. The qPCR reaction was performed in a total volume of 12 μl in each well containing 6 μl of SYBR Green PCR Master Mix (Applied Biosystems), 5 μl of genomic DNA (2 ng/μl), and 0.5 μl of forward and reverse primer each (10 μmol/l). The PCR conditions were as follows: initial denaturation step at 95 °C followed by 40 cycles of denaturation at 95 °C for 15 s and annealing with elongation at 60 °C for 1 min). All reactions were run in triplicate. Target sequences were normalized to albumin (ALB) and, to further assure reliability of the assay, sex determination was performed in reference to factor VIII (F8) located on the X chromosome. The gene copy number was determined with the comparative DDCt method using normal healthy control DNA as a calibrator.

### Breakpoint sequencing

The exact breakpoints of the rearrangement were determined with the use of PCR with primers designed to amplify the DNA fragment spanning the 3′ and 5′ ends of the duplication. PCR was performed in a total volume of 10 μl containing 1 μl of Buffer I (PCR Expand Long Template PCR System v.24; Roche), 0.6 μl of primers (10 μmol/l each), 0.35 μl of dNTPs mix (10 mM), 1 μl of genomic DNA (150 ng/μl), 0.15 μl of DNA polymerase (PCR Expand Long Template PCR System v.24; Roche), and 6.4 μl of PCR-grade water. The PCR conditions were as follows: initial denaturation step at 94 °C for 2 min followed by 10 cycles of denaturation at 94 °C for 10 s, annealing at 60 °C for 30 s, and elongation at 68 °C for 2 min), another 25 cycles of denaturation at 94 °C for 15 s, annealing at 60 °C for 30 s, and elongation at 68 °C for 140 s, and final elongation at 68 °C for 7 min. The PCR product was then sequenced using dye-terminator chemistry (kit v.3, ABI 3130xl) and run on an automated sequencer, Applied Biosystems PRISM 3700 DNA Analyzer.

### *KAL1* relative expression

In order to measure the expression level of the *KAL1* gene in our index case, we performed the relative expression analysis for *KAL1* in blood samples of the proband and seven controls. Total RNA was extracted from peripheral blood mononuclear cells (PBMCs) of the proband and control samples according to the method of Chomczyński and Sacchi (1987). PBMCs were separated from whole blood in a density gradient of Ficoll and sodium diatrizoate (Histopaque®-1077; Sigma). RNA samples were reversely transcribed into cDNA using a mix of oligo-dT and random primers (QuantiTect Reverse Transcription Kit; Qiagen), according to standard protocols provided by the manufacturer.

Quantifications of target and reference genes in cDNA samples were carried out by fluorometric real-time PCR using a LightCycler 480 instrument (Roche Diagnostic GmbH, Mannheim, Germany), a real-time PCR kit (LightCycler® 480 Probes Master), and Universal Probe Library (UPL) probes. For each individual cDNA sample, Cp values were determined in triplicate. The assay was performed using a 96-well plate setup with a final volume of 10 μl/well, consisting of 0.5 μM of each gene-specific primer, 0.1 μM of the appropriate UPL probe, 5 μl of LightCycler® 480 Probes Master mix, 1 μl of the cDNA template, and PCR-grade water. The PCR protocol consisted of an initial denaturation at 95 °C for 5 min, followed by 45 cycles of amplification consisting of denaturation at 95 °C for 10 s, annealing at 55 °C for 30 s, and elongation at 72 °C for 10 s.

Relative expression values of *KAL1* were obtained using the LightCycler480 Software by determining the ratio between the target and two reference transcripts (*ACTB*, *HPRT*). Error bars represent the target/reference error calculated by the LightCycler480 Software in a single experiment performed in triplicate. Relative expression values were normalized to the mean of the controls (K1–K7).

### Fusion transcript relative expression

The relative expression value of the *KAL1* fusion transcript in the proband was determined using the comparative DDCt method by intra-sample normalization to the endogenous control gene *PBGD* and inter-sample normalization to the *KAL1* wild-type transcript, used here as a calibrator. The experiment was carried out with the use of a ViiA™ 7 Real-Time PCR System (Applied Biosystems) and SYBR Green PCR Master Mix (Applied Biosystems). The qPCR reaction was performed using a 96-well plate in a total volume of 12 μl in each well containing 6 μl of SYBR Green PCR Master Mix (Applied Biosystems), 1 μl of cDNA, 0.5 μl of forward and reverse primer each (10 μmol/l), and 4 μl of PCR-grade water. The PCR conditions were as follows: initial denaturation step at 95 °C followed by 40 cycles of denaturation at 95 °C for 15 s and annealing with elongation at 64 °C for 1 min). All reactions were run in triplicate. Primers for the target sequence were designed to specifically amplify the fusion transcript and not the wild-type form.

## Results

### Clinical report

The proband, a 12-year-old boy of Polish ethnicity, was born by spontaneous delivery after an uneventful pregnancy (G1P1) at 41 weeks and 3 days of gestation to a non-consanguineous and healthy 21-year-old mother and a 24-year-old father. At birth, his weight was 2,980 g (3rd−10th percentile), length 51 cm (50th percentile), head circumference 32 cm (below the 3rd percentile), and his Apgar score was 5, 7, 9, and 10 at 1, 3, 5, and 10 min, respectively. Physical examination after birth revealed bilateral malformation of the feet composed of hypoplasia and syndactyly of the postaxial toes, which could have been categorized as ectrodactyly. Additionally, the left hand showed a supernumerary digit, which was fused with the index finger. Transfontanellar ultrasound performed after birth and at day 26 revealed bilateral intraventricular hemorrhage (IVH) of the first degree, whereas abdominal ultrasound was normal. X-ray scan showed fused processus spinosus of L4 and L5. Hearing tests done at 9 months of age showed profound left-sided hypoacusis and normal right-sided hearing. Ophthalmologic examination was unremarkable. At the age of 3 years, the patient was operated on the hydrocele of the left testis, as well as cryptorchidism of the right testis. According to anamnesis, motor development was normal, with independent sitting and walking achieved on time (6 and 12 months, respectively). Expressive speech was delayed, with the first several words at 4 years of age. Due to congenital defects and delayed speech at that time, the proband was referred for a conventional chromosomal analysis (performed on peripheral blood lymphocytes with a resolution of 550 bands per haploid genome), which showed normal male karyotype (46, XY). Psychological assessment performed at the age of 8 years according to the WISC-R test showed 59 points in the verbal scale, 82 points in the non-verbal scale, and 67 points in the full scale. In addition, at the same time, the patient was diagnosed with attention deficit hyperactivity disorder (ADHD). Throughout the childhood, the boy manifested increased sensitivity to smell. Upon repeated brain magnetic resonance imaging (MRI) scans, a cyst of septum lucidum 5 × 26 mm in size was noted. At the age of 11 years and 6 months, the body measurements were as follows: height 141 cm (25th percentile), weight 50.0 kg (75th−90th percentile), and body mass index (BMI) 25.25 (90th−97th percentile). Bone age assessment based on left carpal X-ray was relevant to the metrical age.

**Fig. 1:**
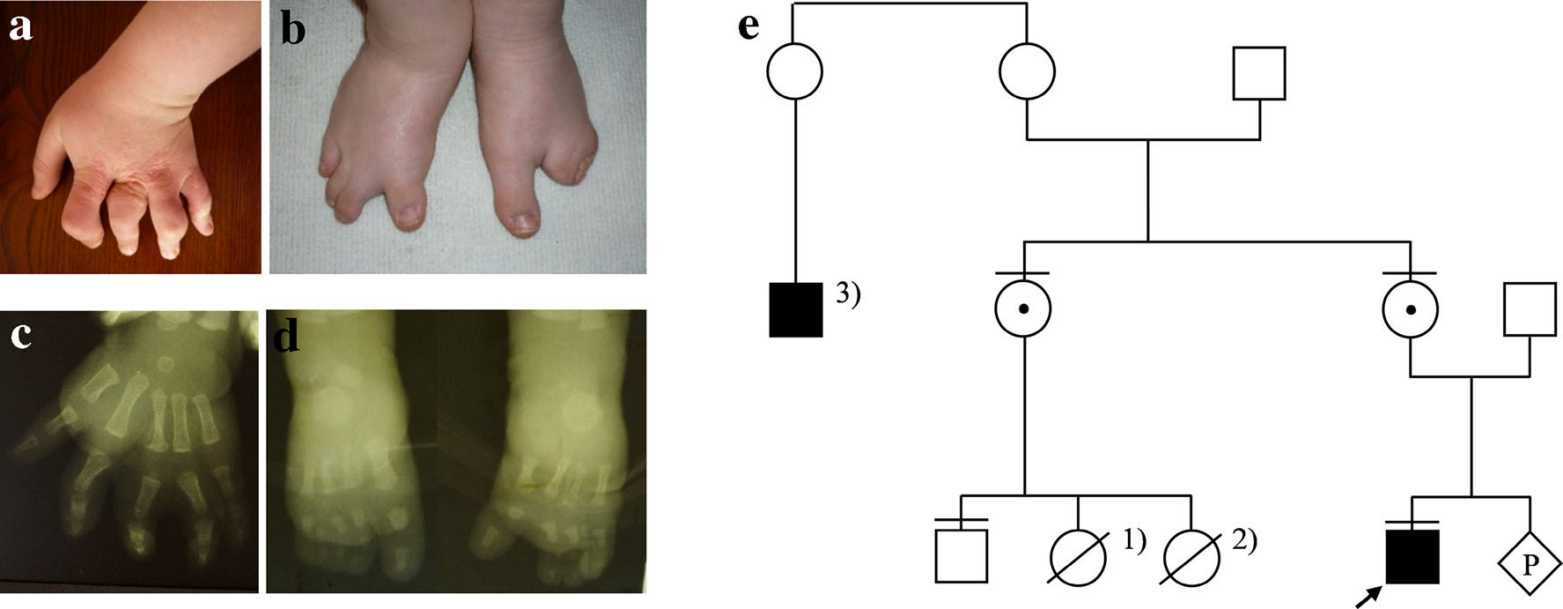
Limb phenotype of the proband and his family tree: **a**, **b** clinical and radiological presentation of the malformation observed in the left hand, **c**, **d** clinical and radiological picture of the ectrodactyly noted in the proband’s feet, **e** pedigree tree showing that the index case is the only affected member of the family. The mother of the proband and maternal sister are the healthy carriers of the duplication. Legend for other genetic conditions occurring within this family: *1* gastroschisis, *2* lethal osteogenesis imperfecta, *3* Down syndrome

The boy was referred to our genetic clinic for diagnosis and first investigated at the age of 12 years. Upon examination, he presented with stocky build, obesity, steatomastia, small penis, and craniofacial dysmorphic features comprising round face, full cheeks, hypotelorism, up-slanted palpebral fissures, and hypoplastic alae nasi (no consent for publication of the full-face photo). His upper limb malformation was bilateral and comprised hypoplastic middle and distal phalanges of fingers 2–5 (as shown in Fig. 1a, b). Lower limb malformation could be categorized as ectrodactyly and was composed of hypoplasia and syndactyly of toes 2–5, which had rudimentary or absent middle and distal phalanges. In addition, both feet showed severe hypoplasia of one digital ray (Fig. 1c, d). Due to the reported increased sensitivity to smell, we decided to perform objective olfactory analyses. While quantitative olfactory examination was symmetrically normal, the registration of olfactory evoked potentials (OEPs) showed considerable increase of the amplitudes symmetrical on both sides. Upon stimulation with peppermint oil and anise oil, the OEP amplitudes were elevated 20 times for nerve I (N1 = 320 ms) and 15 times for nerve V (N5 = 320 ms), showing extreme olfactory hypersensitivity in comparison with the reference values. Endocrinological tests done at 12 years of age showed normal results of TSH, fT3, fT4, FSH, LH PRL, and ACTH. Night release of growth hormone (GH) was unremarkable. Luteinizing hormone-releasing hormone (LH-RH) stimulation test showed normal levels of FSH, LH, and testosterone. The family tree of the proband is presented in Fig. 1e.

### Array CGH

**Fig. 2:**
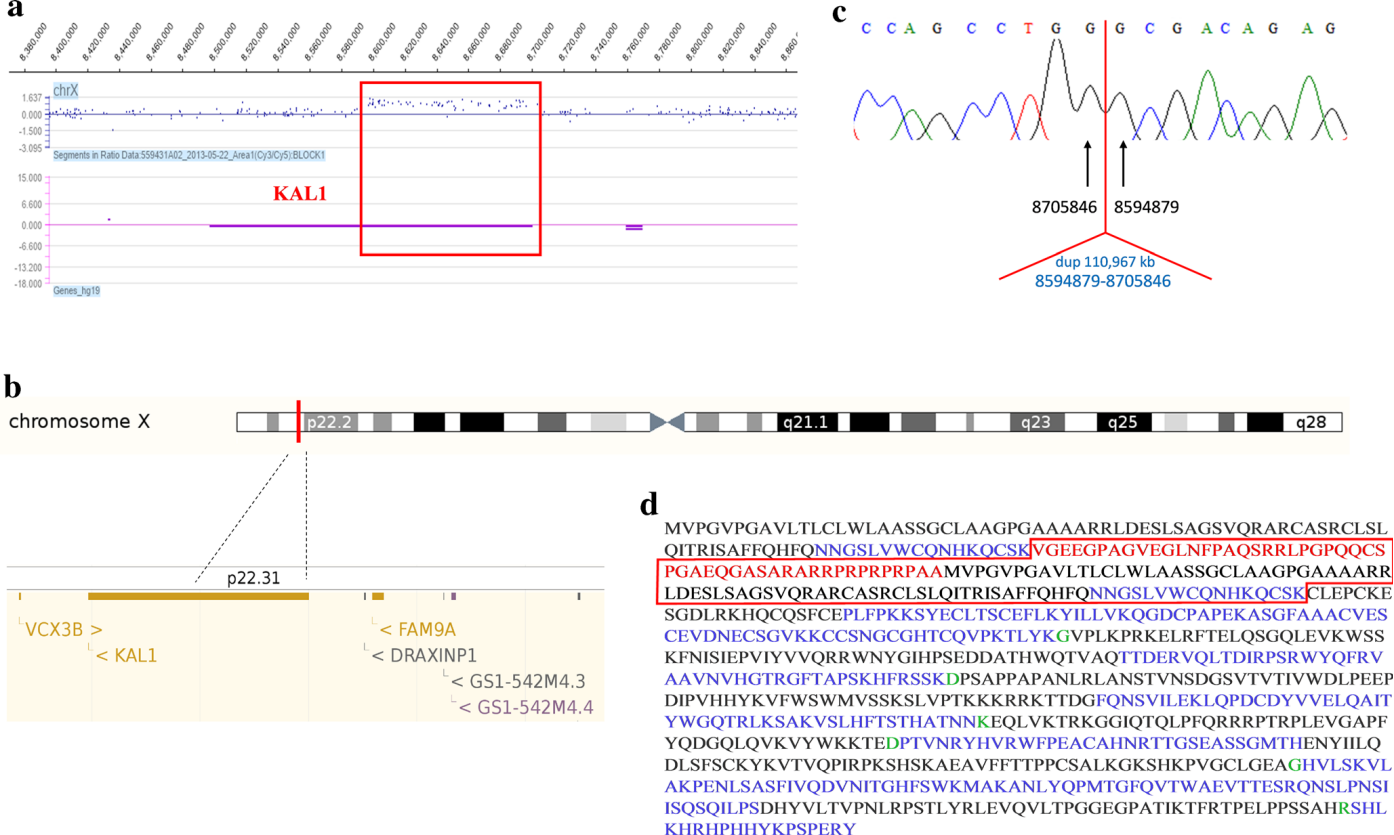
**a** Ideogram of the array comparative genomic hybridization (CGH) results; the *red box* indicates the duplicated region encompassing the 5′ fragment of the *KAL1* gene. **b** Schematic representation of the microduplication in the Xp22.31 locus. **c** Breakpoint sequencing result: a 110,967-kb duplication, with genomic coordinates located between 8594879 and 8705846 according to HG19. **d** Amino acid sequence of the *KAL1* fusion protein; the *red box* indicates the duplication including the translated fragment of 5′-UTR (marked in red color) and the first two coding exons (highlighted by black and blue colors, respectively). The green color indicates amino acid residues encoded by the exon–exon junctions. (Color figure online)

Array CGH performed in the index and subsequently his mother revealed an interstitial microduplication in the Xp22.31 locus, with a minimal size of 108,316 bp (genomic coordinates 8596840–8705155 according to HG19; shown in Fig. 2a). The duplication encompassed exclusively the promoter region and the first two exons and introns of the *KAL1* gene (for a schematic representation, see Fig. 2b) and was inherited from the clinically unaffected mother.

### qPCR and breakpoint analysis

First, the qPCR assay was carried out with a set of five primer pairs in the proband and his mother, in whom it confirmed the array CGH results. Next, other rounds of qPCR done in the proband allowed to narrow down the duplication region, thereby enabling us to design primers for breakpoint sequencing. The exact breakpoints were established by means of PCR with primers involving centromeric and telomeric ends of the microduplication, followed by Sanger sequencing of the PCR product. The exact size of the duplication was 110,967 bp, with genomic coordinates according to HG19 located between 8594879 and 8705846. The breakpoint spanning sequences are shown in Fig. 2c. Finally, we performed segregation studies in all available family members, i.e., unaffected maternal sister and her healthy son. Using qPCR (five primer pairs), we demonstrated that the maternal sister was a heterozygous carrier of the duplication, whereas her son had a single copy of the tested region.

### Fusion transcript analysis

In silico prediction suggested the possibility of the production of two distinct *KAL1* transcripts in our proband. In addition to the wild-type transcript, a fusion transcript containing an insert of 405 nucleotides derived from the duplicated *KAL1* segment could have been expressed. cDNA sequencing of *KAL1* showed the expression of both forms in our proband. The fusion transcript is predicted to give rise to a fusion protein containing 135 additional amino acids at the 5′ end (for the sequence of the putative fusion *KAL1* protein, see Fig. 2d).

### *KAL1* relative expression

**Fig. 3:**
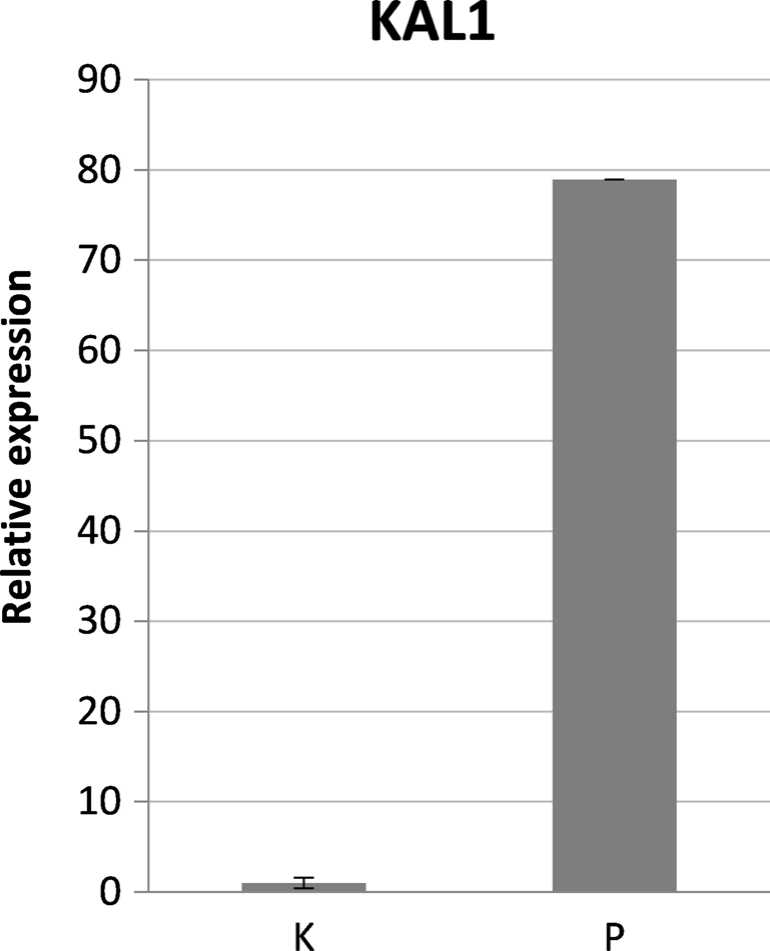
Bar graph showing the relative expression of *KAL1* in the proband in reference to the mean value of seven healthy controls. The expression of the *KAL1* transcript in the proband is 79 times higher compared to the controls

The analysis of *KAL1* relative expression performed in blood samples of the proband and seven controls revealed 79 times higher expression level of the *KAL1* gene in the proband compared to the mean value of the controls (Fig. 3). The relative expression of the fusion *KAL1* transcript in our proband referred to the total pool of *KAL1* mRNA was 0.3 %, suggesting highly preferential expression of the wild-type *KAL1* form.

## Discussion

In this paper, we report on a sporadic male patient presenting with SHFM and profound hyperosmia, accompanied with additional symptoms involving mild intellectual disability, unilateral hearing loss, cryptorchidism, small penis, stocky build, and facial dysmorphism. Since the patient did not show any known recognizable genetic syndrome, we performed high-resolution whole-genome array CGH in order to test for possible imbalanced submicroscopic genomic rearrangement. In the proband, we detected a hemizygous interstitial duplication of 110,967 kb in the Xp22.31 locus, which encompassed the promoter region and the first two exons of the *KAL1* gene. The structural variation showed tandem orientation and was subsequently identified in heterozygosity in the healthy mother of the index case.

The *KAL1* gene is expressed during human embryonic development in the spinal cord, kidney, olfactory bulb, and retina (Duke et al. 1995). Its gene product, anosmin-1, represents a secreted extracellular heparin-binding cell adhesion glycoprotein that controls axonal pathfinding and development of olfactory nerves, as well as migration of gonadotropin-releasing hormone (GnRH) producing neurons to the septohypothalamic area (Cariboni et al. 2004). Loss-of-function mutations of *KAL1*, such as point mutations or deletions of variable size, have been associated with an X-linked form of KS, accounting for 10–12 % of KS cases. The disease is characterized by HH due to GnRH deficiency and anosmia or hiposmia resulting from olfactory bulb dysgenesis. In addition, other anomalies such as sensorineural deafness, renal aplasia, cleft lip/palate, and skeletal defects are known to occur (Dodé et al. 2003). To our knowledge, neither partial nor complete microduplications of *KAL1* have been described in the literature. Consequently, the phenotype associated with such CNVs remains unknown. Therefore, since our proband carried a partial duplication of the 5′ end of the *KAL1* gene, we tested the expression level of this gene in order to confirm pathogenicity of the CNV. Based on blood lymphocyte studies, our proband showed around 80 times higher expression of the *KAL1* gene compared to healthy controls. Unlike KS patients usually affected by hiposmia or anosmia, our proband presented with hyperosmia, which very likely resulted directly from the overproduction of *KAL1* during embryonic development. The mechanism by which *KAL1* is overexpressed in our proband due to the duplication of its 5′ end remains obscure. One possible explanation is that the duplication encompasses yet unidentified *KAL1* enhancer(s), which, by acting in cis in two copies, drive significantly stronger expression of the target gene. Another explanation assumes that, due to the duplication of the promoter region, a putative *KAL1* repressor becomes unable to occupy a newly emerging locus, which, in turns, leads to the repressor insufficiency and increase in target gene expression.

The product of *KAL1*, anosmin-1, is known to directly stimulate tyrosine kinase activity of the fibroblast growth factor receptor 1 (FGFR1), an important signaling molecule involved in a wide range of developmental processes. Loss-of-function mutations of the *FGFR1* gene are another cause of KS identified in approximately 10 % of cases (Dodé et al. 2003). The *FGFR1*-dependent form of KS is inherited in an autosomal dominant manner and frequently presents with limb anomalies such as fusion of the fourth and fifth metacarpals, oligodactyly, and clinodactyly (Jarząbek et al. 2012; Dodé et al. 2003). The role of *Fgfr1* in embryogenesis was established in mouse developmental studies, in which conditional *Fgfr1* knockout organisms manifested cerebral and limb anomalies (Tole et al. 2006; Li et al. 2005; Verheyden et al. 2005; Yu and Ornitz 2008). Decreased *Fgfr1* expression or its complete biallelic inactivation in telencephalon prevents the formation of commissural tracts due to the loss of axonal midline crossing (Tole et al. 2006). In addition, conditional *Fgfr1* knockout mice show aplasia of digits in the central and anterior rays of the autopods, as well as osseous syndactyly of digits III and IV (Li et al. 2005; Verheyden et al. 2005; Yu and Ornitz 2008). These observations point to the importance of *Fgfr1* in limb bud and nervous system formation, and explain the link between insufficient FGFR1 activity and several human congenital malformations, such as agenesis of corpus callosum, hypoplasia of olfactory bulbs, holoprosencephaly, ectrodactyly, or other limb defects.

It was shown that anosmin-1 stimulates the signaling activity of FGFR1 receptor IIIc isoform via direct binding of its N-terminal region to FGFR1 ectodomains (Hu et al. 2009). On the contrary, at high levels, anosmin-1 can also exert inhibitory effects on the FGFR1 protein (Hu et al. 2009; Ornitz 2000). Therefore, in light of these findings, the overexpression of *KAL1* might mimic haploinsufficiency of FGFR1 receptor. Interestingly, *FGFR1* loss-of-function mutations have been shown to give rise not only to KS but also to several other human genetic conditions, such as isolated HH or Hartsfield syndrome, a disorder characterized by the co-occurrence of holoprosencephaly and ectrodactyly, in association with developmental delay/intellectual disability, agenesis of corpus callosum, genital anomalies, and variable skeletal malformations (Simonis et al. 2013). Due to significant overlap in the clinical presentation of our proband and Hartsfield syndrome, as well as common biological pathways involved in the development of both conditions, we hypothesize that ectrodactyly, developmental delay, speech delay, intellectual disability, small penis, cryptorchidism, and facial dysmorphism observed in our proband result from the indirect inactivation of FGFR1 due to significant overexpression of *KAL1*. Of note, there was no evidence for holoprosencephaly upon brain MRI in our patient; therefore, he could not have been diagnosed as having Hartsfield syndrome.

To conclude, in this report, we showed for the first time that increased *KAL1* expression may lead to hyperosmia with significantly increased amplitudes of olfactory evoked potentials. Additionally, we postulate that the partial overlap between the phenotype of our patient and Hartsfield syndrome (including ectrodactyly, intellectual disability, and genital anomalies) may reflect the common pathogenic mechanism (i.e., disturbed *FGFR1* signaling) underlying both conditions. Finally, we hypothesize that the phenotype of our proband represents a novel, probably rare, X-linked recessive congenital malformation syndrome with ectrodactyly as a limb feature, although further reports are needed in order to confirm our assumptions.
